# Medicine

**Published:** 1905-06

**Authors:** George Parker


### Progress of the Medical Sciences.

### MEDICINE.

The prevention of apoplexy is a subject of special interest for us since Professor Allbutt's recent address (vol. xxiii., p. 1), and his previous writings as well as other causes have led to much work on the subject. He excluded from his discussion some important types, such as the occlusion of cerebral vessels by embolism or by thrombosis, whether this latter is due to vascular or hæmic changes. It is hard to say whether there are more cases of healthy lives crippled from the occlusion of cerebral vessels or from the hemorrhage which alone he discusses, but little fresh light has been as yet thrown on the prevention of cerebral thrombosis and its diagnosis while in a remediable stage. Further studies in hæmatology and simple methods for measuring the coagulability of the blood are urgently needed if the vast numbers of hemiplegias of this class are to be reduced. On the other hand, we are indebted to Professor Allbutt for sound teaching on the necessity of recognising early stages of persistent high pressure, of measuring it by reliable instruments from time to time, of removing the causes where they are known, and of preventing a further rise if possible. He points out that there are many cases of high pressure where Bright's disease is not present, that arterio-sclerosis is not a cause but a result of these pressures, and that early treatment will check or prevent the tendency in many cases until the age when danger becomes less or ceases altogether.

One of the most interesting of recent papers on the subject is that of H. W. Cook, late of Johns Hopkins.[1] He lays down that **chronic hypertension** is quite distinct from and much more important than arterio-sclerosis, though either affection may lead to the other. "Apoplexies" occur most often in strong men with nearly normal vessels, but with hypertension, while far older men and women with vessels like beaded pipe-stems are rarely attacked. Between the ages of 45 and 65 forty-three per cent. of the cases occur, and in some families for generations two-thirds of the males regularly die before 60 from this hypertensive diathesis. Hence the importance of treatment before the strain leads to sclerosis, kidney failure, dilated heart, or ruptured cerebral vessels. What are the causes of this chronic high tension? (1) Cook agrees with Clifford Allbutt that it is rarely produced by sclerosis, except in some few cases where the small arterioles are affected. Neither does high tension often lead to true sclerosis, but rather to tortuous and

[1] *J. Am. M. Ass.*, 1905, xliv. 263.

somewhat thickened arteries. (2) Actual causes are certain kinds of lung disease such as emphysema, but not phthisis; (3) some forms of compensated incompetency of cardiac valves, at least during systole; (4) certain brain conditions, and what is very common (5) cardiac hypertrophy from excessive labour or exertion, as in worn-out athletes. (6) The excessive use of tobacco when inhaled is, he thinks, an important factor in raising tension permanently, though it does not primarily cause sclerosis. (7) Alcohol, on the other hand, does not produce hypertension directly, though it may lead to sclerosis, or indirectly, as in alcoholic nephritis, it may cause the production of toxins which act as vaso-constrictors. Probably the direct action of alcohol has been greatly exaggerated, and this view is confirmed by the researches of Cabot,[1] who finds that it does not often cause arterio-sclerosis. Out of 283 cases of severe chronic alcoholism under 50 years of age only 6 per cent. showed changes, and in 95 autopsies where sclerosis was present only 21 per cent. gave histories of alcoholism. In other investigations Cabot[2] found that alcohol caused no definite or permanent rise of pressure when given in various febrile disorders. Indeed, the well-known vaso-dilator action of alcohol would prevent any such result, unless the temporary effect differs utterly from the permanent one. (8) Certain toxins have a vaso-constrictive action, such as those in nephritis, eclampsia, diabetes, and lead poisoning. Others act through structural changes in the vessels, and among these are gout, syphilis, and possibly lead, typhoid and some other febrile disorders, the importance of which Thayer pointed out recently. (9) Lastly, there are many primary or idiopathic cases of high tension of unknown causation. Thus there is an hereditary type, there is Allbutt's plethoric individual who eats huge meals, there is the chronic dyspeptic who generates his own toxins. Constipation in large eaters may be a cause, but there is a constitutional constipation in some persons which has no such effect. Some of Sir George Humphry's centenarians and many poorly-fed persons defecate seldom, perhaps because their digestion is so good that there are few toxins or decomposition products to stimulate the bowels, and in them the tension is low. Mental anxiety and strong emotion have a powerful influence on pulse tension, hence the prevalence of disease in financiers, politicians, and medical men.

J. Barr[3] believes that among causes of high tension we should place an excessive production of adrenalin, and a deficient action of the thyroid, which should counterbalance each other. Another factor is an excess of the purin bodies in the system, and in treatment he would suggest the use of iodides combined with thyroid extract.

Stengel[4], speaking rather perhaps of arterio-sclerosis than of

[1] *J. Am. M. Ass.*, 1904, xliii. 774. [2] *Am. Med.*, 1904, viii. 31.
[3] *Brit. M. J.*, 1905, i. 53. [4] *Am. Med.*, 1904, vii. 9.

simple high tension, holds that its premature development is due to overwork, over-eating, or over-drinking, the absorption of toxins in certain diseases, or of certain metals such as lead. It occurs in the most active and vigorous members of society rather than in the drones. The cardiac and pulse symptoms may precede albuminuria by a long interval, and he agrees with Allbutt's view that the process may often be arrested by reducing the work, the food, and the drink, and by the use of mercurials and salines, and of occasional courses of arsenic and the nitrites. It is said that the bad effects of heavy labour are rarely seen where the work ceases during the winter months, but they are most marked where, as in iron foundries, it is carried on all through the year.

*The symptoms* of hypertension as Cook[1] notices are at first indefinite, such as lassitude, headache, irritability, and shortness of breath, then vertigo, cardiac dilatation, or a trace of albumin, or apoplexy itself may appear. The systolic hypertension varies from 20 to 100 per cent. above normal, the arteries often pulsate visibly, become slowly thickened and tortuous, as Sansom says, an ample dicrotic wave indicates degenerate changes rather than true muscular hypertrophy. The cardiac sounds should also be considered, but it may be difficult to determine whether cardiac hypertrophy exists. In the earlier stages the urine is of high specific gravity, and shows a heavy deposit of urates. Later on the gravity becomes lower, and there may be a trace of albumin. Anderson[2] similarly remarked that in assurance work among business men a good indication of commencing changes was the presence of urine of high specific gravity, hyper-acid, and showing faint traces of albumin and a few hyaline casts with deposits of uric acid or oxalates, an excess of indican and a deficiency of phosphates.

It has been said that not only is the test of the pulse by the finger unreliable, but that the sphygmometer is so also. C. J. Martin[3] shows that this is untrue, and that the resistance of the vessel walls, even in advanced sclerosis, is unimportant, for it only amounts in health to about 2 mm. and in disease does not exceed 7 mm. He finds, indeed, that instruments which apply pressure over a superficial artery, such as the small one of Hill and Barnard and that of von Basch are unsatisfactory and misleading, but better results are given by those which encircle the limb on the Riva Rocci principle. Provided the band is wide enough and the ends of the bag meet, the true internal tension can be determined within 4 per cent. A reliable sphygmometer of this type can be obtained for twenty-five shillings. Cook and Biggs, to whom we owe the elaborate researches carried on at the Johns Hopkins Hospital,[4] strongly recommend an instrument made on the same principle as cheap, and giving an accurate measure of the tension. Sihle,[5] indeed,

[1] *Loc. cit.* [2] *Am. Med.*, 1904, vii. 426. [3] *Brit. M. J.*, 1905, i. 865.
[4] *J. H. Reports.* [5] *Wien. klin. Wchnschr.*, 1904, xvii. 379.

thinks that to get an accurate estimate of the pressure over a large area we should not rely on observations upon a single vessel, and proposes as a test the difference in pressure between the brachial and digital vessels. Possibly this is true when sclerosis is advanced, and he states that the greater the sclerosis the greater the difference between these two pressures, provided that the heart is still strong. Taking the normal difference as 30 to 40 mm., he regards a difference of more than 60 mm. as showing the presence of arterio-sclerosis in the deeper vessels. However, the more important matter is to detect hypertension at an early period before marked sclerosis exists, when the condition can be remedied. For this purpose the use of a handy sphygmometer for clinical work is all-important if we are to avoid the fallacies of ordinary observations on the pulse. Thus if the pulse is "small," that is if the difference between diastolic and systolic pressures is minute, and especially if the heart's action is rapid, we are almost certain to estimate the pressure by the finger as much lower than it actually is.

*The treatment* must of course vary with the cause of the condition. For immediate relief Cook recognises the value of nitroglycerine, but prefers sodium nitrite. Robins finds some efficacy from injections of Blondel's lactoserum. Oliver[1] mentions that ammonium hippurate in 2-grain doses will produce a fall lasting for twenty-four hours. Lichenin, leucin, and tyrosin are also vasodilators, and Trunecek's serum has been already noticed. The reduction of the amount of food taken and the labour undergone are in many cases all important. "The regimen and the waters of certain spas," as Professor Allbutt says, are invaluable. Venesection has but a temporary effect, but free purgation and sweating a more prolonged one. H. Richardson finds some value in thyroid extract and Nauheim baths,[2] while Romberg thinks that iodide of potassium diminishes the viscosity of the blood cells. In the high tension of Bright's disease Hale White finds benefit by reducing the fluid taken, and forbidding exercise, alcohol, meat, and meat extractives, and using free purgation, while Broadbent, who regards uræmic symptoms, and especially convulsions, as largely due to high tension and not to toxins, thinks that bleeding and small repeated doses of mercury are there most efficacious.[3] When the heart has been hypertrophied by overwork, the question arises whether we should rely only on vasodilators, or whether for a time some cardiac sedative such as aconite or veratrin should be employed as well. At least this view has found some support in America.

**Cerebro-spinal meningitis** has at last invaded the daily newspapers which warn us in glowing terms of a coming epidemic

[1] *Lancet*, 1903, i. 1643. [2] *Med. News*, 1903, lxxxiii. 348.
[3] *Brit. M. J.*, 1904, ii. 886.

under the name of "spotted fever." The name is also applied to a disease like typhus produced by a tick in some parts of the States, notably in the Rocky Mountains. The petechial spots, however, are less commonly seen in Europe than in America, where numerous epidemic outbreaks have occurred during the last century. At the present time New York is undergoing a severe visitation, some 1,800 deaths having already taken place there, while eight years ago Boston and the neighbourhood were similarly visited. These outbreaks are not marked by any great differences from the numerous ones on the Continent of Europe; but the mortality in any outbreak is most uncertain, and may vary to an extraordinary degree. While epidemics have followed one another for a century on the Continent and America, sporadic cases only have appeared in Great Britain, though slight outbreaks have been noted in Ireland, in barracks at Dublin for instance. This tendency to appear in crowded places such as barracks has been often remarked, though the disease is not apparently infectious. In spite of careful investigations by Flexner, Councilman, and others, there are endless unexplained points about the disease, partly owing to the low vitality of the organism usually found and the difficulty of growing it. Doubts have been expressed whether this micrococcus intracellularis is always the active cause of the disease, or whether, as in the case of dysentery, other organisms may produce similar symptoms; but Bettencourt and França,[1] in Portugal, found it in all the 271 cases they investigated, while Osler in 35 cases found it in all but three, which were chronic, and in which it had probably died out. It appears as a diplococcus within the polynuclear cells, or free in the spinal fluid, and may form tetrads or clumps but never chains. It is stained by ordinary dyes, but not by Gram's method. Intraperitoneal injections are fatal to guinea-pigs and mice, but rabbits and monkeys seem immune. It is interesting to note that the disease called pink-eye in horses, or epizootic lymphangitis, as our Local Government Board calls it,[2] is due to the same organism, and according to Waitzfelder, epidemics of the two diseases often occur in the same locality.[3] This writer records remarkable success in the treatment of this form of meningitis by the use of diphtheria antitoxin. Besides cardiac stimulants, lumbar puncture was employed to relieve the pressure symptoms, and large doses of antitoxin were injected as soon as possible, and repeated daily in the more severe cases. As much as 10,000 units were used as a dose for adults. The delirium, or coma, more or less disappeared in two days, and the pulse and temperature fell towards normal, while no bad symptoms developed as a result

[1] *Ztschr. f. Hyg.*, 1904, xlvi. 463; abstract in *Med. Chron.*, 1905, 4th ser., viii. 310.

[2] See placards along the country roads of this district.

[3] *Med. Rec.*, 1905, lxvii. 361.

of the antitoxin. In support of his view he refers to an observation of A. T. Wolf that cultures of the meningo-coccus are killed when mixed with anti-diphtheritic serum. Stockton[1] reviews the ordinary methods of treatment, such as Aufrecht's hot bathing, Quincke's spinal puncture and drainage with or without intra-spinal injections of antiseptics, and Angyan's subcutaneous injections of corrosive sublimate; but he has to confess that the reported results are unsatisfactory. Hot baths, spinal puncture, or antipyrin all give great relief, and the same may be said of opiates and bromides; but the mortality is not markedly reduced, though as it varies in different epidemics this is not easy to estimate. As an instance of this Councilman mentions that in the four Massachusetts epidemics the rate ranged from 20 to 75 per cent. The course of the disease varies, too, with individuals. F. J. Love recognises (*a*) a fatal fulminant type which runs its course in a few hours, or at most two days; (*b*) a typhoid one; (*c*) an intermittent type which may last for months, with severe symptoms every day or second day; (*d*) abortive forms which begin severely, but mend after perhaps forty-eight hours; (*e*) mild cases, unrecognisable except during epidemics. The absence of Kernig's sign does not exclude a positive diagnosis, as it cannot be found in some otherwise well-marked cases.

GEORGE PARKER.

